# Supplementary material for: Optimal minimal residual disease threshold in pediatric acute myeloid leukemia: A retrospective cohort study based on the TARGET database
Source: PLoS Med. 2026 May 8;23(5):e1005088. doi: 10.1371/journal.pmed.1005088 (PMC13155632; doi:10.1371/journal.pmed.1005088)
Supplement: S1 Code — (ZIP) [file pmed.1005088.s002.zip › S2 code/PROJ8_14_tbl1/PROJ8_14_tbl1_0.pdf]

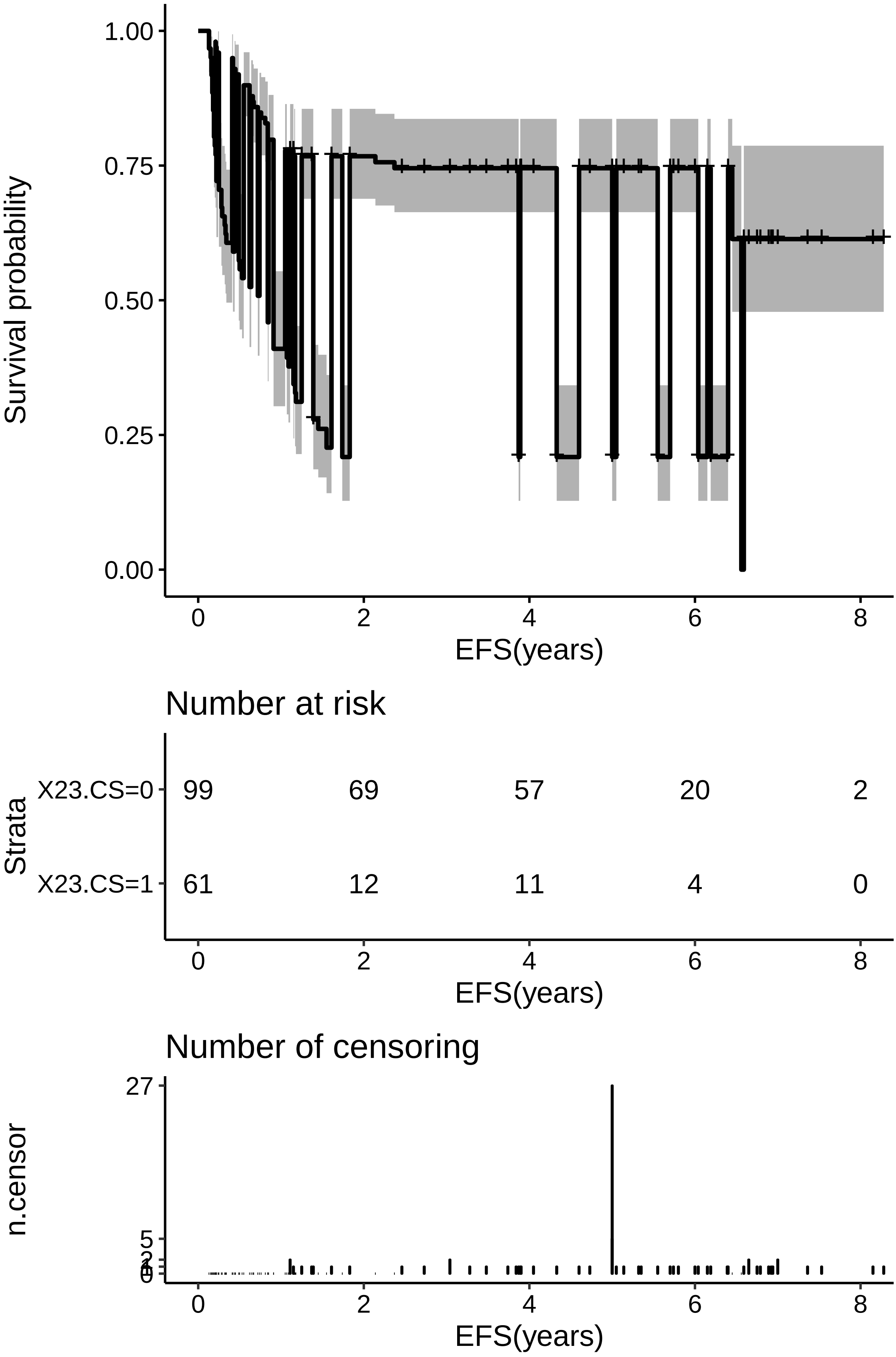

Survival probability
1.00
0.75
0.50
0.25
0.00
0
2
4
6
8
EFS(years)
Number at risk
Strata
X23.CS=0
99
69
57
20
2
X23.CS=1
61
12
11
4
0
0
2
4
6
8
EFS(years)
Number of censoring
n.censor
27
5
2
1
0
0
2
4
6
8
EFS(years)
